# Interdisciplinary education and *authentic development*

**DOI:** 10.1007/s11159-020-09879-2

**Published:** 2021-01-07

**Authors:** Alberto Ciferri, Angelaurelio Soldi

**Affiliations:** 1 Duke University, Durham, NC USA; 2 North Carolina Central University, Durham, NC USA

**Keywords:** authentic development, social harmony, education, wealth distribution, interdisciplinarity, gross domestic product (GDP)

## Abstract

Whereas economists do not traditionally address social harmony, and sociologists or political scientists do not usually tackle economic development, the interaction of social harmony and economic development has recently become an object of intense concern. In their aim to foster evolved rather than uprooted cultural values, the authors of this research note suggest an educational approach to promote the concept and the implementation of what they refer to as *authentic development*. They propose interdisciplinary courses that include notions of history, sociology and economy. Their methodology is based on two main resources: (1) a textbook providing a broad historical survey tracing the development of 28 nations in the Americas; and (2) analytical parameters related to the extent of social interaction and income generation.  Students carry out digital and group research and elaborate the conditions that generate social harmony, economic well-being and a better balance between them. The authors have already piloted their educational approach in four secondary schools in Antigua, Guatemala with 50 students aged 17–19. Most of them are now enrolled at local universities, but the impact of this course on their performance will only emerge over time.

## Résumé

Éducation interdisciplinaire et développement authentique – Bien que les économistes ne se consacrent pas traditionnellement à l’harmonie sociale et que les sociologues ou les politologues ne se penchent pas normalement sur le développement économique, l’interaction entre harmonie sociale et développement économique est depuis peu devenue un sujet de grande préoccupation. Dans leur démarche visant à encourager davantage des valeurs culturelles ayant évolué que des valeurs culturelles déracinées, les auteurs de cette note de recherche suggèrent une approche éducative pour promouvoir le concept et la mise en œuvre de ce qu’ils appellent le développement authentique. Ils proposent des cours interdisciplinaires englobant des notions d’histoire, de sociologie et d’économie. Leur méthode s’appuie sur deux ressources principales: (1) un manuel comportant une vaste enquête historique retraçant le développement de 28 nations des Amériques et (2) des paramètres analytiques liés à l’étendue de l’interaction sociale et la création de revenus. Les étudiants exécutent des recherches numériques et de groupe et élaborent les conditions qui génèrent l'harmonie sociale, le bien-être économique et un meilleur équilibre entre eux. Les auteurs ont déjà expérimenté leur approche éducative dans quatre écoles secondaires guatémaltèques à Antigua, avec 50 élèves âgés de 17 à 19 ans. Si la plupart d’entre eux étudient à présent dans des universités locales, les effets de ce cours sur leurs résultats ne se feront jour qu’avec le temps.

## Introduction

The first of the United Nations Sustainable Development Goals (SDGs) aims to “end poverty in all its forms everywhere” (UN 2015, p. 14). While all 17 SDGs address pressing problems, the fact that poverty is given extra prominence as the first goal is telling. Sadly, the issue is an ongoing one, with economists and sociologists still trying to find the magical solution to solve it. The onset of the COVID-19 pandemic crisis is going to exacerbate poverty dramatically for many people in all world regions.

### Economists

From the time of British economist and philosopher Adam Smith (1981 [1776]) to the most recent Nobel Prize winners, economists have emphasised problems and possible solutions for the reduction of poverty. Tried-and-tested strategies have included microcredit,^1^ social enterprises^2^ and family planning (birth control). Nobel Peace Prize laureate Muhammad Yunus founded a microcredit organisation in 1976 that helped many women in Bangladesh to build well-being by granting them small loans. Yunus also coined the term *social business,* which is similar to the idea of social enterprise (Yunus 1999, 2009). Economist Nobel Prize laureate W. Arthur Lewis was concerned with “the growth of output per head of population” (Lewis 2003 [1955], p. 9). In 2003, Abhijit Banerjee and Esther Duflo founded the Abdul Latif Jameel Poverty Action Lab (J-Pal) at the Massachusetts Institute of Technology (MIT) to study poverty. Unequal distribution of resources is one of the elements that hinder development. Human development theorist Denis Goulet observed that:

a comprehensive ethic of authentic development looks to the sustainable use of [all] resources as well as to their equitable distribution (Goulet 1996, p. 199).

As he built his development theory (and his proposed new discipline of *development ethics*), Goulet drew on philosophy, policy practice and anthropology.

### Sociologists

Sociologists have also greatly contributed to development projects aiming to improve economic well-being (Genovesi 2013 [1802]; Pankaj and Dorji 2004; Sachs 2005). In a book published in 1802, Italian philosopher Antonio Genovesi suggested that culture and civilisation are necessary to promote socio-economic well-being (Genovesi 2013 [1802]). After conducting a survey attempting to measure individual happiness in relation to gross national happiness (GNH) parameters in Bhutan, Prabhat Pankaj and Tshering Dorji (2004) conclude that their findings suggest that even though “income perhaps does add to happiness […] it is a weak variable” (ibid., p. 387). Jeffrey D. Sachs (2005) identified possible solutions to poverty that afflicts different nations in terms of economic, social and environmental problems. Politicians such as Robert F. Kennedy affirmed, already in 1968, that gross national product (GNP)^3^ measures everything except that which is worthwhile (Kennedy 1968). Indeed, there is now widespread consensus that the main component of human happiness is social harmony, followed by economic well-being.

### Behavioural economics

The connection between social harmony and economic development has become an object of intense concern. Religious and political authorities, as well as academics, have discussed the conflicts between the traditional aspirations of society and the pressure for economic growth of the capitalist system, often associated with a deterioration of some democratic processes (Levitsky and Ziblatt 2018). The recent escalation of migratory processes is one macroscopic example of the struggle between economic well-being and ethnic traditions. New development models aiming to bridge the narrow outlooks from economic and social disciplines have emerged. These models span from rational adoptions of concepts typical of the two disciplines to the development of interdisciplinary courses combining economic and social sciences for the education of young generations. The field of *behavioural economics,* for example, pursues a “method of economic analysis that applies psychological insights into human behaviour to explain economic decision-making” (OUP n.d.).

Some of the more radical approaches were chastened by the work of reformists such as Banerjee and Duflo (2012), who proposed a model tailored to the lives and expectations of the poor, arguing that a lot of anti-poverty policies had failed because of an inadequate understanding of poverty, of what it really means to be forced to live on less than 99 cents per day. Luigino Bruni and Stefano Zamagni (2017) proposed a “civil economy” that emphasises principles of responsibility, reciprocity and redistribution over capitalist market approaches, particularly competitiveness and pursuit of profit that have eroded the moral economy. Hunter Lovins et al. (2018) introduced “natural capitalism”, based on business that aims to profit but behaves responsibly towards nature and people, promoting reinvestment in natural capital. Dirk Philipsen (2015) criticised the emphasis on gross domestic product (GDP) which ignores quality, purpose, sustainability and quality of life. Courses and even degrees in “Economic and social sciences” are already being offered (e.g. at Bocconi University in Milan, Italy).

### Authentic development

The concept of a *socio-economic culture* based on a solid historical perspective has not been emphasised in the above models. What we present in this research note is our own concept for the successful promotion of harmony and stability within today’s multi-ethnic communities which does require *an understanding of the events and the struggles* that *caused the creation of new cultures.* We derive this insight from our experience with development projects in Central and South America which have led us to conclude that a new discipline, *authentic development,*^4^ should be promoted for the education of new generations. This new discipline should be taught through and based on principles conducive to achieving harmonious economic development paced to social development, able to mitigate ethnic and class conflicts and foster evolved, rather than uprooted, cultural values.

To achieve these goals, a history book entitled *An overview of historical and socio-economic evolution in the Americas* was elaborated (Ciferri 2019).^5^ This book reviews the most visible socio-economic features of 28 nations in the American continent, and its main objective is to stimulate an appreciation of history and cultural identities (ibid.). The book does not take any particular cultural perspective, neither Euro-Western nor Indigenous. In its historical survey of the Americas, it emphasises pre-Columbian cultures and societies. In the part covering post-Columbian states, it emphasises socio-economic development promoted by conquerors and colonists. The disruptions and changes of pre-existing cultures and the problems faced by modern nations are emphasised. Unpacking complex issues and describing the events that caused the creation of multi-ethnic societies allows the book’s readers to appreciate the evolution of national identities, the ongoing struggles of present populations, and conceive alternative development models.

A course using this textbook has already been piloted in four secondary schools in Guatemala. The course is based on a framework which allows students to arrive at semi-quantitative assessments of various parameters controlling socio-economic development (Ciferri 2018a). The course’s interdisciplinary approach is briefly described in the next section. The course’s particular relevance is highlighted by religious and political authorities’ current call for a need to develop new educational models as the world emerges from the COVID-19 disaster.

## Interdisciplinary education

Interdisciplinary degree programmes are not uncommon in today’s academic institutions, and both the advantages and the shortcomings of interdisciplinary education have been discussed in the literature (Nissani 1977; Newell 1994; Szostak 2009; Jones 2010).

Interdisciplinary study allows for synthesis of ideas and characteristics from many disciplines. At the same time, it addresses students’ individual differences and helps to develop important, transferable skills (OU 2019).

If our students were to follow three different but parallel courses in economics, sociology and history, they might, in sum, gather a multidisciplinary education rich in information within these three disciplines, but they would not be prompted into considering their interactions.

The concept of development has indeed evolved from the notion of economic growth into the idea of economic development which has more recently expanded to include socio-economic development (Litwinski 2017). The intrinsically interdisciplinary nature of development studies and research has been recognised (Molas-Gallard et al. 2014) and online resources for evaluating socio-economic development have been produced (EC 2013). Building on these concepts and resources and expanding their scope, we are the first to propose an interdisciplinary approach based on notions of history, sociology and economy for the benefit of young generations (Ciferri 2018a, 2019). Our interdisciplinary approach involves students in what is referred to as *deep learning:*

Cognitive scientists think of deep learning – or what they might call “learning for understanding” – as the ability to organize discrete pieces of knowledge into a larger schema of understanding […] another aspect of deep understanding [is that it] requires both a significant repository of factual knowledge and the ability to use that factual knowledge to develop interpretations, arguments, and conclusions (Metha and Fine 2019, p. 12)

Indeed, traditional teaching has been practised under the paradigm, or assumption, that it consists in the transmission of knowledge from an authority, the teacher, to the students. But, as argued by Brazilian educator and philosopher Paulo Freire,

Liberating education consists in acts of cognition, not transferrals of information. It is a learning situation in which the cognizable object (far from being the end of the cognitive act) intermediates the cognitive actors – teacher on the one hand and students on the other (Freire 2000 [1970], p. 79).

Teaching should aim to develop independent analytical thinking in learners and even inspire them to produce original work. The students in our pilot courses worked in groups, and they were taught to synthesise historical, social and economic outlooks.

## Methodology of the course

### General scheme

The aim of the course we conceptualised is to stimulate the interest of new generations of students in the origin of current conflicts, poor wealth distribution and the excesses of capitalism. We initiated our course in Spanish because our experience with development projects in Central and South America revealed an immediate need for initiating an intervention there. Over the past three years, we have already piloted this course with teenage students in four private secondary schools in Central America (Guatemala).^6^ We are currently exploring possibilities of extending it to tertiary-level/higher education institutions (HEIs) in both Spanish-speaking and English-speaking locations.

In our pilot courses, we adopted the following sequence of classes

- Introductory classes using preparatory material on subjects discussed in the book that the students might not have previously studied (e.g. forms of governments, ethnic groups, religions, economy).
- Classes on the nations of the Americas (featuring dedicated individual chapters of the book) which may be covered during a semester, with an emphasis on ethnic and social class conflicts and economic resources.
- “Laboratory” sessions introducing the students to semi-quantitative parameters for the characterisation of social harmony, economic well-being and socio-economic balance for each of the selected nations. (These parameters are discussed in more detail in the next section).^7^
- Elaboration by the students of socio-economic development models relevant to a particular country using concepts and analogies derived from the study of several chapters of the book and other literature, often on the internet.
- A final exam in which each student is asked to describe the current situation of a particular country and her/his own tentative model for its socio-economic development.

In Table 1, we detail the sequence of classes taught in our first pilot course during the second semester of 2017 at the Fray Luis de la Cruz Education Centre, a private secondary school in Antigua, Guatemala.

**Table 1:** Schedule of the General Culture course for fourth- and fifth-year students of the Science and Literature Baccalaureate offered at the Fray Luis de la Cruz Education Centre in Antigua, Guatemala (26 June–19 November 2017)

Week	No. of days	No. of hours	Topic
26–30 Jun	1	1	INTRODUCTION
10–16 Jun	2	2	Propaedeutic [introduction to the field of]: CULTURE
17–23 Jul	3	3	Propaedeutic: ETHNICITY
24–30 Jul	1	1	Propaedeutic: POLITICS
01–06 Aug	2	2	Propaedeutic: RELIGION
07–13 Aug	2	2	Propaedeutic: ECONOMY
21–27 Aug	0	0	——————
28–31 Aug	2	2	Propaedeutic: ECONOMY
04–10 Sep	1	3	Mid-term EXAM
11–17 Sep	2	2	USA / Parameters
18–24 Sep	2	2	MEXICO+ GUATEMALA/Parameters
25–30 Sep	0	0	——————–
02–08 Oct	2	2	NICARAGUA/Parameters
09–15 Oct	1	1	COSTA RICA/Parameters
16–23 Oct	2	2	CUBA/Parameters
23–29 Oct	0	0	——————-
06–12 Nov	3	3	DEVELOPMENT MODELS
12–19 Nov	1	3	Final EXAM
*Total*	*27*	*31*	

*Notes: n* =12 (5 female, 7 male) students; age = 17–19 years

Teachers: Celeste Velasquez (History), Jaime Psiquiy (Religion), Nino Hernandez (Sociology), Yordy Gabriel Noriega Moron (Economy)

The other chapters of the textbook (Ciferri 2019) were covered in the next semester (January–June 2018)

### Introductory classes

The purpose of the introductory classes was to familiarise students with the basic cultural, social and economic principles discussed in the textbook which they might not have previously studied. These classes are meant to develop the students’ general cultural awareness and to prepare them for conducting, in their next steps, analyses of the ways in which these principles developed in each American country.

### The classes on the nations of the Americas

The classes on the nations of the Americas involved the study of selected chapters of the textbook focusing on the way in which ethnic and class conflicts and economic resources affected the development of particular countries. The teacher would emphasise any possible correlation between the history of a particular country and its present socio-economic situation.

### Definition and use of the parameters

**Fig. 1:**
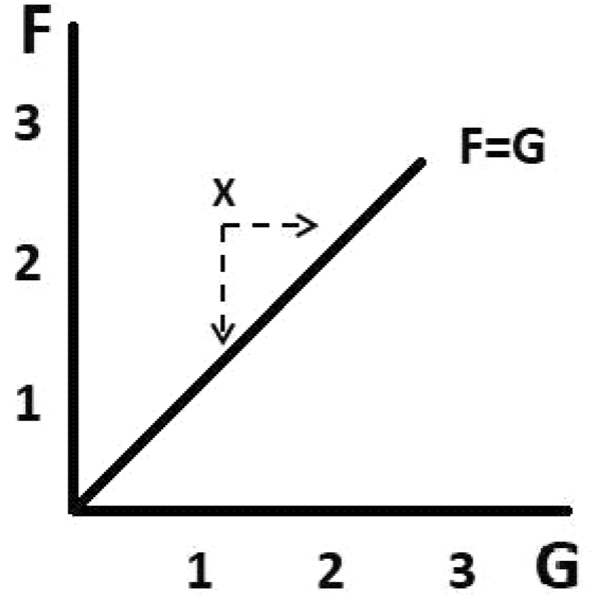
Relationship between *F* (the extent of social interaction) and *G* (the extent of income generation). The *F*=*G* line represents the ideal balance between the socio-economic parameter (*F*) and the economic well-being parameter (*G*). The efficacy of development models is gauged by the separation between the ideal line and the *F*/*G* ratios (e.g. *x*) evaluated with the qualitative approach

The purpose of the laboratory sessions was to let the students discover semi-quantitative parameters for the characterisation of social harmony, economic well-being and socio-economic balance for each of the selected nations. An integral part of our approach is the analysis of a parameter (*F*) to express the extent of social interaction, and a parameter (*G*) to express the extent of income generation. The ratio of these two parameters (*F*/*G*) represents the balance between social harmony and economic well-being (Ciferri 2018a), with *F*=*G* constituting an ideal balance (see Figure 1).

In order to perform a quantitative evaluation of these parameters, we might follow an approach similar to that used by sociologists Pankaj and Dorji (2004) for the evaluation of happiness. Thus, we could attempt to dissect and evaluate the complex variables affecting *F* and *G*, and assume a common dimensional unit as done by Pankaj and Dorji (ibid.). However, while each nation’s *G* parameter can be assumed to mainly reflect that country’s corresponding GDP, we feel that expressing *F* as a function of several, supposedly independent, variables would be problematic. Thus, for a preliminary analysis of both parameters, we opted for the adoption of a less ambitious, more qualitative approach which assigns the (dimensionless) numeric values of 1, 2 and 3 respectively to insufficient, average and high estimates of either *F* or *G*.

In the laboratory sessions of our pilot courses, the students assessed the value of *F* by group discussions following the study and comparison of selected chapters on the book, eventually integrated with information from related work (Pankaj and Dorji 2004; Banerjee and Duflo 2012; Philipsen 2015; Bruni and Zamagni 2017; Lovins et al. 2018). The data and information they used for their assessment of *F* were limited to (1) the *strength of family ties,* (2) *treatment of the elderly* and (3) *social conflicts*. The assessment of *G* was based only on GDP data collected in the book.

While the qualitative nature of the above analysis needs to be emphasised, it should not be underscored. Our aim is not to give to the students established formulas to calculate socio-economic parameters to be used for viable development models. Rather, our aim is to *develop a culture that emphasises a balance of human values and economic well-being* within the historical perspective offered by the textbook. The evaluation of the relationship *F*/*G* between the two parameters has primary significance in our attempt to consider and express a guideline for new development models. The value *F/G* ~ 1 (or *F*=*G*, see Figure 1) represents a well-balanced society, assumed as a reference state (note that for a balanced society to be a *productive* one, both *F* and *G* ought to be > 1). A society in which excessive weight is given to economic development (e.g. the United States [USA]) corresponds to *F*/*G* < 1, and a society where social harmony prevails over economic development (e.g. Guatemala) corresponds to *F*/*G* > 1. It is to be stressed that *both rich and economically depressed countries need to adjust their F and G parameters to approach the situation of authentic development F/G ~ 1.*

### Elaboration by the students of socio-economic development models

Table 2 shows the data the students in our first pilot class assessed for different nations using the above qualitative approach.

**Table 2:** Examples of students’ estimated values of the parameters

Country	*F*	*G*	*F*/*G*
USA	2	3	0.6
Guatemala	3	1	3
Chile	2	2	1
Haiti	1	1	1

*Notes: F*= extent of social interaction; *G*= extent of income generation; F/G=ratio of the two parameters (i.e. extent of socio-economic balance)

The values represent 1 = insufficient, 2 = average, and 3 = high estimates

The students’ evaluation is not surprising. While the USA scores highest for *G* (based on GDP), Guatemala scores highest for *F.*

### Final exam

In the final exam at the end of the course, each student is asked to describe the current situation of a particular country, proposing alternative development models on the basis of analogies with neighbouring nations.

### Envisaged parameter refinement for university-level students

To elaborate a more quantitative comparison with development in neighbouring countries, we are planning to adopt an original approach towards a *quantitative* assessment of the parameters. Such a quantitative assessment will be based on the assumption that *F* and *G* do not represent a country’s social harmony and GDP, but rather the *percentage rate of change* of either. As fractions, percentages are dimensionless and can be measured on a numerical scale neglecting the different dimensionalities of the factors included in *F* or *G*. A dimensionless numerical value of the cumulative percentage of growth for each year over a range of years is produced by adding the measured percentage growth in *G* and *F* published in each successive year. For instance, *G* may be assumed to change according to predicted (published) alterations that reflect yearly variations of GDP for a particular country. These data, plotted as in Figure 1, will produce a *co-evolution* graph showing how the two parameters keep pace with each other. The distance between the co-evolution graph and the ideal *F*=*G* line can be defined and computed, thus giving a measure of how a proposed or historical development model approaches ideal, authentic development.

The above theoretical framework, which leads to a co-evolution graph, is essentially a visualised and comparative assessment tool meant to achieve two goals: (1) the evaluation of quantitative values of *F* and *G,* and (2) the assessment of how a desirable social development (*F*) keeps pace with economic growth (*G*) over a period of time in different societies and economies. The number of variables we use is limited, since our purpose is to teach how to identify what may balance economies and well-being, rather than aiming to mimic real situations, except for historical studies documenting past developments and thus providing a tool for validating and improving the definition and computation of *F*. Statistical methods would further advance the study of the relevant relationships, as would other tools in economics, social and political science.

### Limitations and weaknesses

The weakest point in the implementation of the latter analysis is the identification of the variables affecting *F* or *G*, including their weighted fraction and their mutual independence. The number of variables affecting *F* does indeed include a large variety of social factors, in addition to those few considered in the qualitative analysis described above. For instance, data on migration, level of education, crime rates, unemployment rates, economic inequality and more, are also relevant in studies of correlations between economic development and social change for particular communities. Information on factors like prejudices, bigotry or racism may be more difficult to come by and therefore accessible only in an approximate sense in terms of the level of literacy or education.

In the elaboration of our envisaged quantitative analysis we shall use a limited number of relevant variables identified by independent studies. The weight of each variable will be expressed by a factor that gives the normalised percentage of people affected by that variable. While our course does not itself teach how to preserve culture, it does encourage students to incorporate their particular cultural background in identifying the relevant components of the *F* parameter. The evaluation of happiness for different countries, which has been extensively reported in the literature, is also affected by the difficulty in assessing all the relevant variables (Pankaj and Dorji 2004).

### Development models

*F*/*G* represents the socio-economic gap between the development in a country and the reference state. The main aim of our course is to inspire students to develop models aiming for the *reduction* of such a gap (*F*/*G* → 1) (Ciferri 2018a, 2019). The analysis of individual countries described in the textbook (Ciferri 2019), complemented by additional research on related work (Pankaj 2004; Banerjee and Duflo 2012; Philipsen 2015; Bruni and Zamagni 2017; Lovins et al. 2018), suggests several strategies for assessing individual countries’ economic and social parameters.

In Guatemala, for instance, the high *F*/*G* ratio of 3 (Table 1) could be reduced by models that lower *F* (e.g. loosening family ties through migration or job mobility), or by independently increasing *G* (e.g. by promotion of economic activities). In the case of the USA, the low value of the *F*/*G* ratio (0.6; shown in Table 1) could be increased by strategies aiming at a reduction of *G* or an increase of *F*. However, in view of the skyrocketing profit economy, which is occupying the mind of a large component of the adult and young population, the former approach might not produce significant results. Therefore, we envision educational models based on the voices of many humanists, artists and immigrants for an increase of the social harmony parameter *F*.

There are some strategies that induce inversely-related changes in *G* and *F*. For instance, job mobility reduces *F* and consequently induces an increase of *G*. These situations allow the alteration of parameters that are not easily evaluated. In the case of the USA described above, direct alterations of *G* may not be easily achieved. However, *mitigating job mobility* will increase *F* and decrease *G*. Related considerations pertain to factors such as *cooperation, punctuality*, *responsibility.* These variables reflect the (intensive) quality of social interaction, and indirectly influence economic activities, thus favouring an increase of *G*. These considerations highlight the role of education in the simultaneous development of social harmony and economic activities.

As indicated above, the aim of our course is a purely educational one, namely the promotion of cultural awareness and the provision of knowledge and tools for creating a more harmonious socio-economic culture for *future* generations. Therefore, the viability of the models for socio-economic development proposed by the students will not be an essential requirement. Moreover, the implementation of *viable* models would encounter serious difficulties, particularly when strong ethnic differences occur. For instance, the cultural incompatibility between Native Americans and European colonists is mainly due to the desire of the former to preserve their traditions and protect the environment for the next generations. Native Americans would not oppose useful innovations and, in our view, could work harmoniously with a US government under a clearer statement of their cultural identity. In Brazil, a lack of conflicting interests has produced a remarkable harmony between various ethnic groups. Nevertheless, explicit discrimination still affects dark-skinned individuals. In similar cases, no amount of education will help socio-economic development unless the *current* administration systems change their colonialist nature, or the economy is willing to share leadership with Black people.

One example of development models considered by our pilot students is the control of the competition between supermarkets and the small family-run “*tiendas”* in rural communities of Central America.^8^ The *tiendas* (*t*) are considered a kind of connective tissue within rural society, and thus make a significant contribution to *F* in addition to having a modest impact on *G* (*F*_*t*_/*G*_*t*_ >1). By contrast, while supermarkets (*s*) do not greatly contribute to *F*, they do contribute to *G* mostly through salaries paid to local employees (*F*_*s*_/*G*_*s*_<1). The strategies for balancing the overall socio-economic parameter *F*_*ts*_/*G*_*ts*_ (with *ts* designating *tiendas* coexisting with supermarkets) considered by the students involved the relatively simple evaluation of the overall monetary parameter *G*_*ts*_ and the analysis of strategies aiming to convince the municipalities and the supermarkets to reduce the competition with the *tiendas*. Other examples of development projects compatible with the preservation of some features of local culture have been considered (Lovins et al. 2018; Ciferri 2019).

## Preliminary results and expectations

**Fig. 2:**
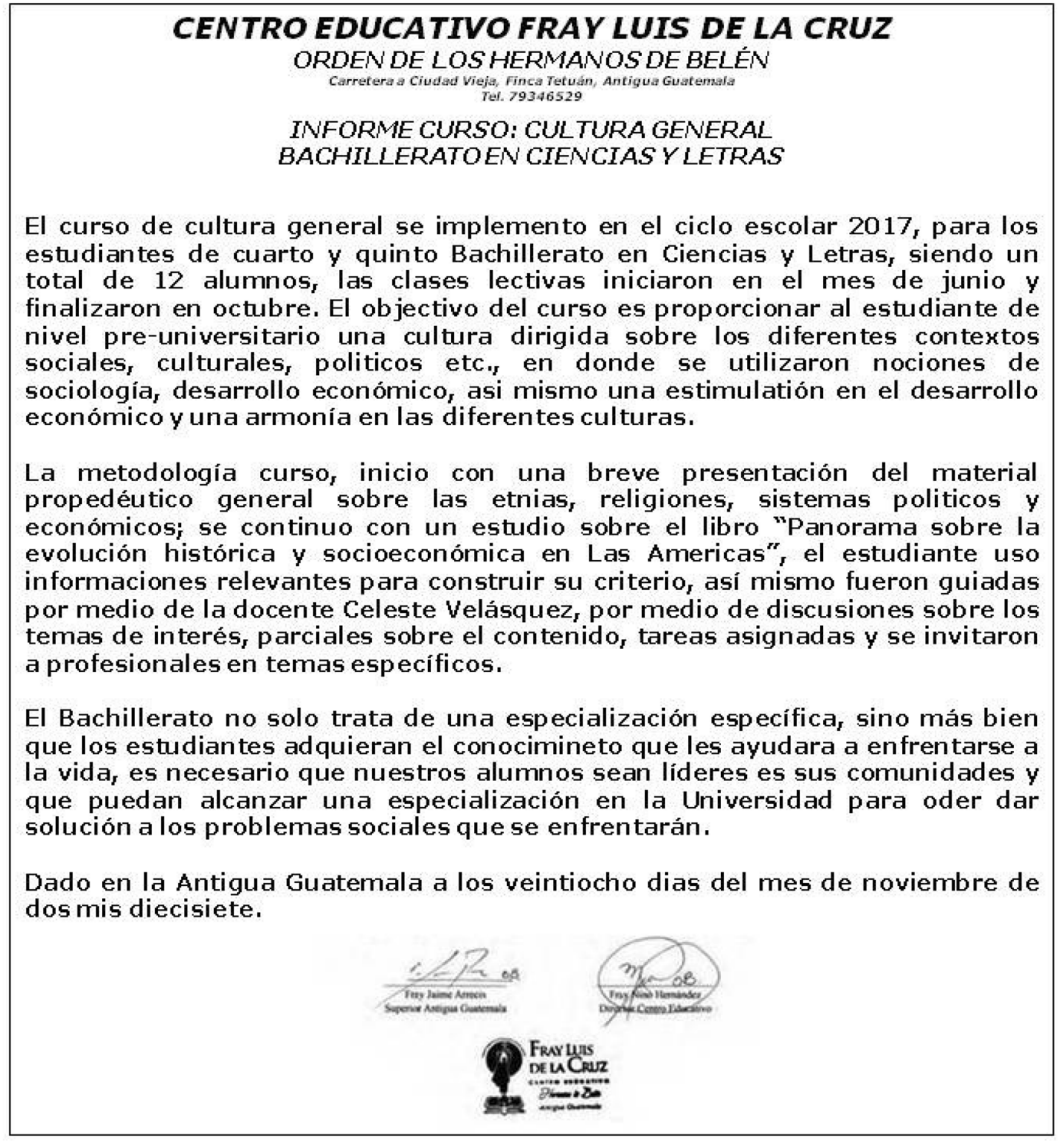
Document regarding the inauguration of the first course offered in Antigua during the second semester of 2017

As mentioned earlier, so far we have run four pilot courses in four private secondary schools in Guatemala. The first course began in Antigua in 2017–2018 (see Table 1). The inauguration message (in Spanish) is reproduced in Figure 2. Here is a rough translation of it into English:

***FRAY LUIS DE LA CRUZ EDUCATION CENTRE***

*ORDER OF THE BROTHERS OF BETHLEHEM*

Carretera a Ciudad Vieja, Finca Tetuán, Antigua, Guatemala, phone 79 34 65 29

Course profile: General Culture

Science and Literature Baccalaureate

The General Culture course was implemented in the 2017 academic year for fourth- and fifth-year students in secondary school preparing for the Science and Literature Baccalaureate. A total of 12 students participated in the course. Classes began in June 2017 and ended in October 2017. The objective of the course is to provide the pre-university student with a cultural outlook directed towards different social, cultural and political contexts using notions of sociology and economic development that stimulate authentic development and harmony in different ethnic cultures.

In terms of methodology, the course begins with a brief presentation of general introductory material on ethnic groups, religions, and political and economic systems found in the Americas. It continues with the study of a textbook, *An overview of historical and socio-economic evolution in the Americas,*^9^ edited by Alberto Ciferri. The students are taught to use relevant information to construct analytical parameters related to the extent of social interaction and income generation. In the 2017 pilot course, students were guided by professor Celeste Velásquez through discussions on the topics of interest, based on teaching materials and assigned tasks complemented by sessions on specific topics taught by invited experts.

The Baccalaureate is not only about a single specialisation, but rather strives for students to acquire the knowledge that will help them to face life, serve as leaders to their communities, and achieve specialised expertise at university so that they can provide solutions to the social problems they face.

Signed in Antigua, Guatemala, on the twenty-eighth day of November of the year two thousand and seventeen: Brother Jaime Arreces, Superior Antigua Guatemala. and Brother Nino Hernández, Director of the Education Centre, Fray Luis de la Cruz Education Centre, Order of the Brothers of Bethlehem, Antigua, Guatemala.

So far, a total of about 50 students have been involved, revealing a remarkable interest in the course, which was offered as an elective component of the Science and Literature Baccalaureate. Most of our course participants are now enrolled at local universities, but the impact of our course on their performance will only emerge over time. Plans to extend the course to first-year university students in the USA and in Guatemala are under way.

The selection of teachers suitable for our interdisciplinary classes is an issue that has required careful consideration. For the propaedeutic classes in history, sociology and economy we invited specialists who were interested in participating in the project. Each chapter, focusing on one American nation, includes a scholarly description of the main historical events and a brief presentation of data on its territory, its ethnic composition and current socio-economic situation. Each chapter also includes a brief insight into how the successes or difficulties of the individual country relate to cultural and historical events and to the evolution of that country’s national identity or, indeed, identities.

We found that university students teaching our course at secondary school level were able to deliver and perform an acceptable synthesis of the notions of history, sociology and economy, provided they had been given the opportunity to carefully study and elaborate the chapters of the textbook, often in cooperation with a voluntary teacher or with the editor of the book.

To set up this course at university level, it would be advisable to establish an interdisciplinary panel composed of a historian, a behavioural economist and a sociologist. They could analyse the book chapters with a view to tertiary study and select the best combination of teachers for each class. Considering that the textbook provides adequate descriptions of historical events, the panel could perhaps even be limited to a sociologist and an economist. Another alternative would be a single professional with experience or degrees in both economics and sociology.

In the long term, the evolution of this project might eventually lead to the establishment of a new interdisciplinary subject that bridges traditional history, sociology and economics courses. *It should also be remarked that* although the present courses were designed for English- or Spanish-speaking students in the Americas, *the approach based on the interdisciplinary focus on redevelopment issues might be extended to other regions*. The project’s sponsor, the Jepa-Limmat Foundation,^10^ would assist any learning institution in the Americas willing to consider the establishment of interdisciplinary socio-economic courses. One distinct outcome of implementing the project’s courses will be the preparation of qualified professionals who can support a balance between social harmony and wealth distribution and the mitigation of ethnic and class conflicts, thus promoting a better functioning of democracy in both developed and developing countries. This long-term aim would be consistent with, or even exceed, the targets for quality education of the fourth United Nations Sustainable Development Goal (SDG 4; UN 2015).

## Footnotes

1 The term *microcredit* is known mainly in the developing world, where it refers to the lending of small amounts of money at low interest to new businesses.

2 A *social enterprise* is an organisation whose main objective is to have a social (economical or environmental) impact rather than making a profit for its shareholders.

3 A country’s *gross national product* (GNP) represents its gross *domestic* product (GDP) plus its net factor income from abroad. Expressed as a monetary value, GNP takes into account all finished goods and services (disregarding intermediate ones) produced by the country, irrespective of where the factors of production (land, labour, capital, and entrepreneurship) are located.

4 Others, in particular human development theorist Denis Goulet, have used the term “authentic development” within the wider context of development ethics (Goulet 1996, 2005, 2006).

5 The textbook was published in Spanish first as an eBook (Ciferri 2018b), and then in English in a hard-cover edition (Ciferri 2019). The table of contents and editorial overview of the English version of the book are available here: http://www.jepafoundation.org/pdf/An-Overview-of-Historical-and-Socio-economic-Evolution-in-the%20-Americas.pdf [accessed 26 October 2020].

6 Public education in Guatemala is free and compulsory between the ages of 7 and 14. Children enter primary school (grades 1–6) at age 7 and secondary school (years 1–5) at age 12. Students in Guatemala complete their formal secondary schooling with the Baccalaureate, which qualifies them for entering tertiary (university) education. The official language of instruction is Spanish, even though it is not all citizens’ first language. Due to infrastructural limitations of Guatemala’s rural areas, there is an abundance of private schools, many of which are run by Catholic religious orders (Jesuits, Marists or Bethlehemites), who also operate some of the country’s hospitals. Our student sample comprised 12 fourth- and fifth-year students who were aged 17–19. Our General Culture course was offered at their school as an elective component of their Science and Literature Baccalaureate, and they participated in our pilot course by choice.

7 Learning in a “laboratory” setting through active experimentation or exploration is not confined to natural sciences and engineering. Laboratory classes can be conducted in any subject. Students are asked to investigate a given topic or conduct an experiment and verify their results, often comparing them with an expected outcome.

8 *Tienda* is the Spanish word for shop.

9 This refers to the Spanish Kindle eBook edition (Ciferri 2018b).

10 The Jepa-Limmat Foundation, associated with the Limmat-Stiftung [Foundation] in Zurich, Switzerland, was legally recognised on 17 February 1995 to continue the activity of the Jepa Foundation, established in Genoa, Italy on 3 June 1994. Their activity is centred in medical and educational projects. The Foundations are running development projects in Guatemala, Venezuela, Mongolia and Kazakhstan that aim to promote a reduction of the socio-economic gap between developed and developing countries. For more information, visit http://www.jepafoundation.org.
